# Subcutaneous Emphysema Caused by an Extraperitoneal Diverticulum Perforation: Description of Two Rare Cases and Review of the Literature

**DOI:** 10.1155/2018/3030869

**Published:** 2018-07-31

**Authors:** Gael Kuhn, Jean Bruno Lekeufack, Michael Chilcott, Zacharia Mbaidjol

**Affiliations:** Hôpital Fribourgeois Riaz, Rue de l'Hôpital 9, Case Postale 70, Riaz, 1632 Fribourg, Switzerland

## Abstract

The onset of colon diverticular disease is a frequent event, with a prevalence that increases with age. Amongst possible complications, free peritoneal perforation with abscess formation may occur. We herein describe two rare presentations of an extraperitoneal sigmoid diverticulum perforation. Our first patient, an 89-year-old female with no signs of distress, developed a subcutaneous abscess and emphysema in an incisional hernia following an appendectomy through a McBurney incision. The second patient, an 82-year-old female, was in general distress at the time of her admission and had a more advanced infection following the occurrence of a sigmoid perforation in a hernial sac. Complicated diverticulitis has a known course and evolution, but with an extraperitoneal presentation, this etiology is not expected. A computed tomography (CT) scan should be completed if the patient is hemodynamically stable, and wide debridement should be performed. Subcutaneous emphysema with an acute abdomen may be a sign of sigmoid perforation. Clinicians should keep this etiology in mind, regardless of the initial presentation.

## 1. Introduction

Diverticular disease is a “Western” disease with a prevalence increasing with age, with 50% to 60% of people over the age of 80 being affected. The distal colon is mostly impacted, with 10% to 20% of patients afflicted with diverticular disease presenting with diverticulitis [1–4]. Perforation of the diverticulum may occur, causing a wide range of complications that are mainly intraperitoneal in nature, such as abscessation, fistulization, and hemorrhage. In rare cases, the diverticulum can perforate in an extraperitoneal manner. In 1853, the first case of a subcutaneous emphysema secondary to the perforation of a hallow viscous was described [5]. Herein, we report two unusual presentations of the extraperitoneal manifestation of a perforated diverticulitis.

### 1.1. Case 1 Presentation

**Figure 1:**
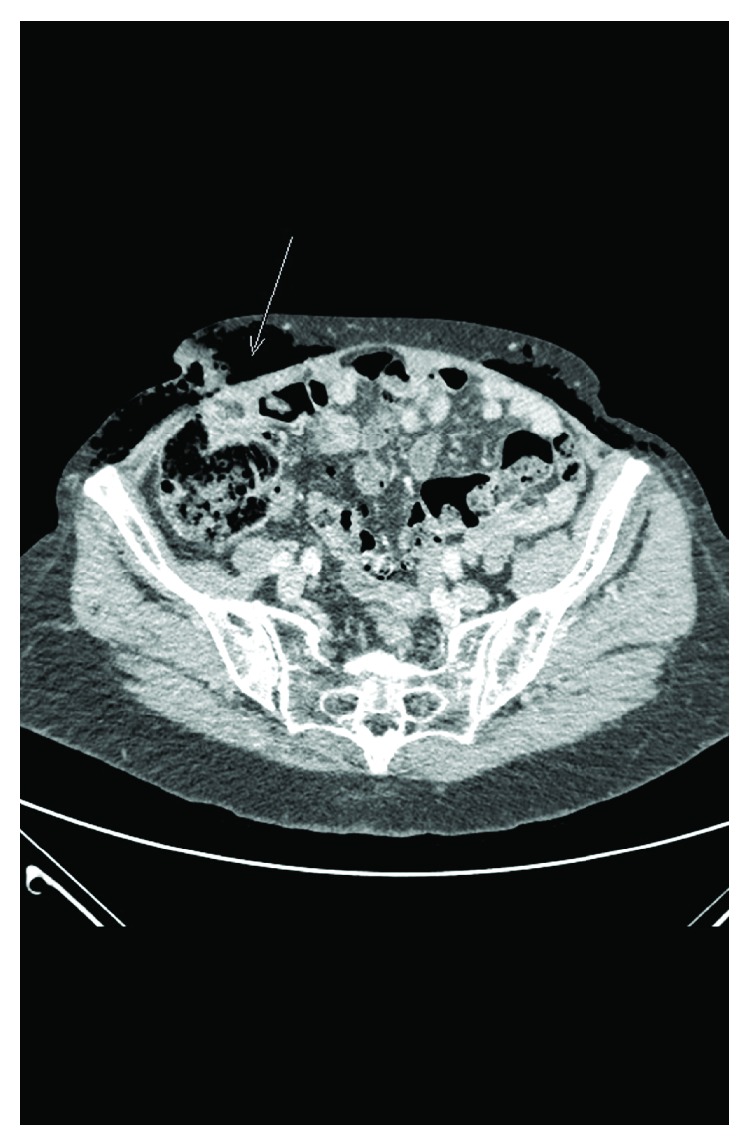
CT findings of the first case. The arrow indicates the extraperitoneal perforation.

An 89-year-old fit female with a history of chronic back pain and an appendectomy during her youth completed using a McBurney incision presented with a one-day history of spontaneous pain in her right flank without any fever, chills, or other symptoms. At the time of her admission, she was not in distress, she was not febrile, and her vital signs were within normal values. On clinical examination, there was swelling with a red area measuring 12 cm × 4 cm and tenderness of the right flank around her appendectomy scar. Crepitus could be felt diffusely on her right and left flanks and the periumbilical and epigastric regions upon palpation. Blood test showed the presence of mild inflammation, with a CRP value of 7 mg/l (within normal values) and an elevated white blood cell count of 18 G/l. The rest of the laboratory results were normal. Emergency ultrasonography was unhelpful because of air interference. An abdominal CT scan (Figure 1) showed diffuse subcutaneous abdominal emphysema extending to the pelvis on the left side that was more pronounced on the right inguinal fossa with a bowel loop in contact with the abdominal wall. An emergency laparotomy centered on the McBurney incision showed feces and pus within the subcutaneous compartment. Furthermore, at the level of the aponeurosis of the external oblique muscle, an inflammatory diverticulum could be seen fistulizing between the lumen of the sigmoid colon loop and the necrotic subcutaneous tissue. We subsequently diagnosed intraoperatively a subcutaneous abscess and emphysema with an enteroparietal fistula caused by a ruptured sigmoid diverticulum in an incisional hernia. The necrotic tissues were excised, and the punctiform sigmoid colon fistula was closed. Revision of the rest of the sigmoid showed important adhesions between the sigmoid colon and the parietal peritoneum of the right flank and between the caecum and the sigmoid colon, respectively. The sigmoid colon also showed diffused diverticulosis with no inflammation. The cutaneous and subcutaneous tissues were left open and dressed with a negative pressure-assisted closure device on postoperative day 1. The patient received intravenous antibiotherapy for two weeks with quinolones and a third-generation cephalosporin at first which was then switched to aztreonam due to an allergic reaction. Bacteriological studies showed polymicrobial digestive bacteria (i.e., *Escherichia coli*, *Streptococcus equinus*, and *Enterococcus*). Subsequently, there was good clinical and biological evolution. At two weeks postoperation, she was reoperated on for closure of the wound. She was discharged from the hospital three weeks after her initial surgical intervention with the indication to continue antibiotics for a total of four weeks.

### 1.2. Case 2 Presentation

**Figure 2:**
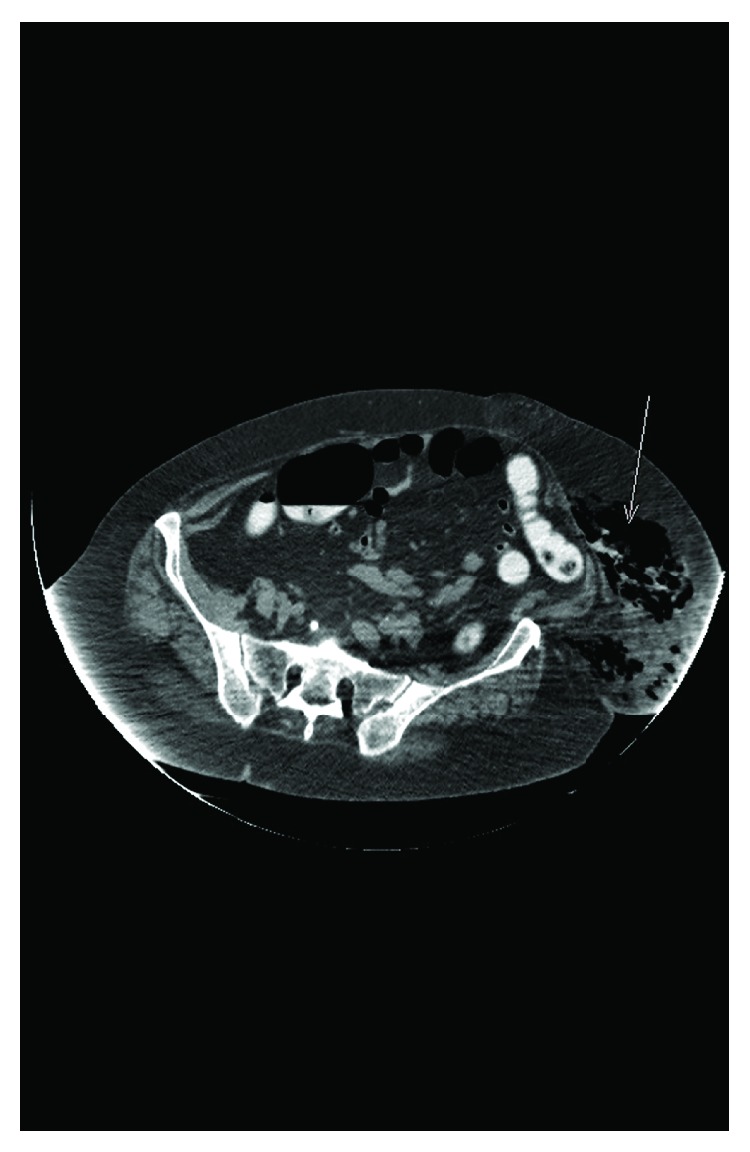
CT findings of the second case. The arrow represents once again the extraperitoneal perforation.

An 82-year-old patient with a history of diverticular disease, a hysterectomy 20 years ago, and a gastric ulcer who had experienced a digestive hemorrhage 18 years ago was admitted to the emergency room with a one-week history of diminished general health associated with abdominal pain, diarrhea, and fever. Her general practitioner reported that she had sustained many falls during the past week and had felt sleepy since the previous day. At the time of her admission, the patient was very weak but had a normal state of consciousness. She had no fever (36.8°), a blood pressure of 80/40 mmHg, a pulse rate of 100 beats/minute, and a room air saturation at 80%. The physical examination showed a woman in general distress with a diffusely sensitive lower abdomen. Her left inguinal fossa demonstrated an important hematoma extending to the left flank (resulting from the various falls) that was in the process of abscessation with phlyctena and a local smell of necrosis. The rest of her abdomen was nontender. A blood test revealed a marked inflammation with a CRP value of 440 mg/l and a white blood cell count of 22 G/l, with a left shift. Her creatinine clearance value was 25 ml/mn. The abdominal X-ray gave an impression of subcutaneous feces on the left flank. An abdominal CT confirmed the presence of a sigmoid perforation, with liquid extending from a suprapubic hernial sac all the way to the subcutaneous tissue on the left abdominal wall, which contained free air and feces (Figure 2).

The diagnosis of septic shock with subcutaneous emphysema caused by a sigmoid perforation was retained, and an emergency surgical laparotomy was suggested. The patient received wide-spectrum antibiotics, and a segmental sigmoid resection was performed with creation of a Hartmann terminal colostomy. Culture swab and biopsies yielded a mixed flora (e.g., *Streptococcus dysgalactiae*, *Klebsiella oxytoca*, *Proteus mirabilis*, *Morganella morganii*, and *Enterococcus*). The necrotic cutaneous and subcutaneous tissues on the left abdominal wall were widely excised, all the way to the muscular fascia from the left flank to the left inguinal fossa. The wound was then partially closed on multiple drains. Twenty-four hours later, she was reoperated upon for more extensive debridement of the soft tissues and the abdominal fascia on the right and left inguinal fossa and the supra pubic region. The wound was then dressed with a negative pressure-assisted closure device and closed on drains located near each parietal colic space. Pathology findings confirmed the diagnosis of severe and perforated diverticulitis with necrosis of the abdominal wall. In the postoperative course, her general status degraded with the development of a cardiorespiratory distress condition requiring intubation and amines. The patient died 12 days later from complications of her disease.

## 2. Discussion

Diverticular disease of the colon is a multifactorial disease influenced by ethnicity, diet, and possibly genetics [2]. It is also an age-related disease affecting less than 10% of people younger than the age of 40 to two-thirds (67%) of the population older than 80 [2–4]. In one study, diverticulum of the descending colon and sigmoid was recorded in more than 5% of postmortem examinations performed in persons aged 40 and older at the Mayo Clinic [1]. The disease has a high prevalence in Western countries. It affects mostly the distal colon, with 90% of cases having a sigmoid involvement and 15% having a right-sided diverticulum [4]. Between 10% and 20% of patients affected with diverticular disease present with diverticulitis [2–4]. Perforation can be localized and cause an abscess, which may be drained under CT guidance or it may perforate into the free peritoneal cavity and cause extensive peritonitis [1]. To our knowledge, there have been only three descriptions of a sigmoid diverticulitis perforation into an inguinal hernia [6–8] and only one previous case of a subcutaneous emphysema with necrotizing fasciitis caused by a sigmoid diverticulitis perforation reported in the literature [9]. Furthermore, we believe that this present case report is the first description of subcutaneous emphysema caused by a diverticulum perforating into an incisional hernia following an appendectomy. In both of our cases, perforation of the diverticulum into the extraperitoneal space occurred via a point of weakness in the abdominal wall. In the first case, it was through an incisional hernia. By definition, these hernias develop at the site at which an incision has been made for a previous surgery, complicate 5% to 11% of wound closures [10], and carry a relatively high mortality rate (5.3%) with complications such as an enterocutaneous fistula, adhesions, and strangulation [10]. In the second case, the sigmoid diverticulum perforated into an inguinal hernia. Salemis et al. described a case of an incarceration of Meckel's diverticulum through a ventral incisional defect [11], while Alvarez-Zepeda et al. reported a case of perforated sigmoid diverticulum in a Spigelian hernia causing a necrotizing fasciitis [12]. Both of these cases are already very rare presentations of diverticulum perforation. Diverticula most commonly form due to a rise in intraabdominal pressure, such as that caused by muscle contractions during straining for defecation. They usually occur on the antimesenteric border, at a point of weakness on the musculature wall of the bowel, mostly between the taeniae coli around the point of entry of the vasa recta [3, 4, 13]. Patients in Western nations mostly present with left-sided involvement of the colon (90%), whereas, in contrast, those in Asian nations show right-sided involvement [3, 4]. Painter and Burkitt suggested that a poor-fiber diet predisposes individuals to diverticular disease by increasing transit time and decreasing stool volume [4, 14] and therefore, diverticular disease is a preventable condition. One feared complication of diverticular disease is perforation with abscess formation. In very rare instances, gas cracklings under the skin can be felt following perforation. Subcutaneous emphysema can be a nonspecific sign of rupture of a hollow organ [5, 13, 15]. Air formation can occur by way of two different mechanisms, either (1) due to the air gradient between the perforated organ with the persistent peristalsis and the subcutaneous tissues or (2) secondary to an infection by gas-forming bacteria, mainly *Escherichia coli*, *Clostridium sporogenes*, *Enterobacter aerogenes*, *Klebsiella*, and *Proteus* [5, 16, 17]. In some cases, perforation of the gastrointestinal tract may drain to the buttock, hip, thigh, or lower extremities or into the retroperitoneal space of the abdomen by dissecting along the anatomical planes [18]. Morton reported a case of a sigmoid diverticulitis perforating into the right buttock, the left buttock, and the hips [1]. In gas gangrene, due to the above-cited microorganisms, the air lies within the muscles, as opposed to that in the case of subcutaneous emphysema, where it lies in between the muscles and interstitial tissues [16]. In our first case, the gas had spread via a complicated diverticulitis, which had caused a colocutaneous fistula and the perforation of a diverticulum in the abdominal wall, possibly secondary to a weakened abdominal wall and previous surgery. During her physical examination, the crackling was extending diffusely on her abdomen, but there were no signs of peritonitis or widespread cellulitis, suggesting a direct spread of gas into the subcutaneous tissues. Furthermore, the bacteria found with the culture swab were organisms that were part of the endogenous flora. The perforation was localized and occurred on the opposite side due to a redundant sigmoid loop. In our second case, which had a fatal outcome, the patient first presented with a cellulitis that evolved into a necrotizing fasciitis of the anterior lateral wall, requiring another surgery. There was also no gas gangrene, but, with the infection being more advanced, the case required a sigmoid resection, extensive necrosectomy, and a Hartmann terminal colectomy. Neither of the two patients were diabetics, but the second patient was obese and had hypertension, chronic renal insufficiency, and chronic venous lower extremity insufficiency, whereas the first patient was in good general heath otherwise. Fecal peritonitis, which has a high mortality rate (46%) [9], was not present in both of the cases we have described, as the feces had accumulated in the extraperitoneal space. CT scanning, which was necessary in both cases to determine the nature of the disease and the extent of the perforation, is the best imaging modality available to clinicians at this time and permits the elucidation of characteristic features for detecting the site of perforation, free gas, or fluid accumulation, allowing for an easier differentiation between air and fatty tissues. The findings can be wall thickening, pericolonic stranding, or a massive pneumoperitoneum [19]. An emergency surgery with extensive debridement and necrosectomy is the usual outcome of such findings, as simple percutaneous drainage is rarely enough and a second operation is often needed. Agaba et al. reported a case of subcutaneous emphysema with muscular necrosis and necrotizing fasciitis from a perforated sigmoid diverticulitis that required two consecutive surgeries [9]. During the operation, it is important for the surgeon to differentiate between a subcutaneous tissue infection with emphysema caused by free air drained in the subcutaneous tissue and necrotizing fasciitis and gas gangrene, as the latter two carry much higher rates of mortality and morbidity. This diagnosis should be considered in patient experiencing exquisite pain with a systemic toxicity, extensive cellulitis, crepitus, and/or hyponatremia [20, 21]. In both of our current cases, the wounds were then closed with a vacuum-assisted closure device very early on, which helps with wound healing by stimulating blood flow, decreasing local tissue edema, and removing excess fluid. Its use also increases cell division, tissue granulation, and bacterial clearance [9, 22]. Notably, despite the administration of prompt and appropriate treatment, the outcome of our second case was fatal. This outlines the difficulty that persists in handling such infections.

## 3. Conclusion

Diverticular disease is a frequent disease with known evolutions and complications. In some rare instances, which we have illustrated herein, complications may occur in a setting that renders the diagnosis more complicated. Extraperitoneal sigmoid diverticulum perforation is an unusual occurrence. Furthermore, crepitus and abdominal subcutaneous emphysema on imagery represent a high index of suspicion and warrant a search for the cause. Aggressive surgical debridement should immediately be performed. A better prognostic outcome can be expected with a prompt diagnosis and when the fascia is not affected, but, ultimately, the end result depends on the patient's comorbidities.
